# The Relationship between the Misfolding Avoidance Hypothesis and Protein Evolutionary Rates in the Light of Empirical Evidence

**DOI:** 10.1093/gbe/evab006

**Published:** 2021-01-11

**Authors:** Dinara R Usmanova, Germán Plata, Dennis Vitkup

**Affiliations:** 1 Department of Systems Biology, Columbia University, New York, NY, USA; 2 Elanco Animal Health, Greenfield, IN, USA; 3 Department of Biomedical Informatics, Columbia University, New York, NY, USA

**Keywords:** protein evolution, molecular clock, protein function, protein stability, protein misfolding

## Abstract

For more than a decade, the misfolding avoidance hypothesis (MAH) and related theories have dominated evolutionary discussions aimed at explaining the variance of the molecular clock across cellular proteins. In this study, we use various experimental data to further investigate the consistency of the MAH predictions with empirical evidence. We also critically discuss experimental results that motivated the MAH development and that are often viewed as evidence of its major contribution to the variability of protein evolutionary rates. We demonstrate, in *Escherichia coli* and *Homo sapiens*, the lack of a substantial negative correlation between protein evolutionary rates and Gibbs free energies of unfolding, a direct measure of protein stability. We then analyze multiple new genome-scale data sets characterizing protein aggregation and interaction propensities, the properties that are likely optimized in evolution to alleviate deleterious effects associated with toxic protein misfolding and misinteractions. Our results demonstrate that the propensity of proteins to aggregate, the fraction of charged amino acids, and protein stickiness do correlate with protein abundances. Nevertheless, across multiple organisms and various data sets we do not observe substantial correlations between proteins’ aggregation- and stability-related properties and evolutionary rates. Therefore, diverse empirical data support the conclusion that the MAH and similar hypotheses do not play a major role in mediating a strong negative correlation between protein expression and the molecular clock, and thus in explaining the variability of evolutionary rates across cellular proteins.

**Significance**

Evolutionary rates vary substantially across cellular proteins. Understanding the nature of the molecular clock and its variability across proteins is a foundational question in molecular evolution. The popular and currently dominant theory to explain the molecular clock variability is the misfolding avoidance hypothesis (MAH). The role and importance of the MAH is currently under active debate. In this article, we discuss how to appropriately test the MAH based on available empirical data. We then rigorously test the hypothesis using more than a dozen new genome-wide data sets that characterize protein stability and aggregation propensities. Our results demonstrate that the MAH is unlikely to play a major role in explaining the variability of the molecular clock across proteins.

## Introduction

Protein evolutionary rates vary by orders of magnitude across cellular proteins, but the mechanisms underlying this variability are currently unknown (Koonin 2012). Although protein expression was shown to be the strongest predictor of protein evolutionary rates across species (Pal et al. 2001, 2006), the causes of the anticorrelation between expression and protein evolutionary rate are not understood (Zhang and Yang 2015). The popular misfolding avoidance hypothesis (MAH) posits that the sequences of highly abundant proteins evolve slowly *primarily* due to increased selection against misfolded protein toxicity (Drummond et al. 2005; Drummond and Wilke 2008; Yang et al. 2010). The recent availability of genome-wide experimental data on protein stability has reinvigorated the debate about the model of protein evolution based on the MAH (Plata and Vitkup 2018; Razban 2019).

We read with interest the recent article “Protein melting temperature cannot fully assess whether protein folding free energy underlies the universal abundance–evolutionary rate correlation seen in proteins” by Razban (2019). This article is related to our previous analyses, that is, Plata *et al.* (2010) and especially Plata and Vitkup (2018). Razban’s study discusses our results showing a lack of empirical support for the MAH based on the genome-wide protein melting temperature ($T_{m}$) data obtained by Leuenberger et al. (2017). To avoid potential misunderstanding in the field, in this article we address inaccuracies in the characterization of our previous work, comment more broadly on the proper usage of experimental data to test the MAH, and then further test the hypothesis using multiple new empirical data sets.

The MAH can be tested by investigating the two key predictions of the hypothesis: 1) protein abundance positively correlates with protein stability (Drummond and Wilke 2008; Zhang and Yang 2015), and 2) protein stability substantially affects the variation of evolutionary rates across cellular proteins. As we demonstrated previously, a careful re-analysis of the proteome-wide $T_{m}$ measurements obtained by Leuenberger et al. (2017) shows no support for the MAH in *Escherichia coli* and three other investigated species (Plata and Vitkup 2018). Razban’s study states that our analysis provides support for the MAH in *E. coli* due to a correlation between protein abundances and melting temperatures. This claim is not correct and, we believe, exemplifies a common and unfortunate confusion. As we specifically discussed (Plata et al. 2010; Plata and Vitkup 2018), the MAH cannot be validated simply by demonstrating a weak correlation between protein abundance and stability, that is, the relationship (1) above. The MAH, at its core, is not only about the stability of highly expressed proteins but also about a major effect of protein stability on the level of sequence constraints across cellular proteins. Thus, it is essential to investigate whether protein stability accounts for any substantial fraction of the variance of evolutionary rates across proteins.

In this study, we analyze available Gibbs unfolding free energies (${\Delta G}^{0}$) for *E. coli* and *Homo sapiens* proteins (Kumar et al. 2006) and multiple $T_{m}$ proteome-wide data sets characterizing protein misfolding and aggregation propensities in several species (Levy et al. 2012; Savitski et al. 2014; Becher et al. 2018; Mateus et al. 2018; Volkening et al. 2019). Notably, ${\Delta G}^{0}$ and $T_{m}$ measurements represent different and complementary approximations for *in vivo* protein stability. Although an advantage of ${\Delta G}^{0}$ is that it is the direct measure of protein stability, an advantage of genome-wide $T_{m}$ measurements is that they represent proxies of protein stability in the natural cellular environment. Our analyses, using these two different measures of protein stability, show no support for a major role of the MAH in any considered organism.

## Results

The central message of Razban’s analysis is that the absence of the expected relationship between $T_{m}$ and protein abundance may be due to an imperfect correlation between measured $T_{m}$ and ${\Delta G}^{0}$ (Razban 2019). Razban evaluated this correlation based on the error model constructed using the *E. coli* data set from Leuenberger et al. (2017). To address the issue of the correlation between protein melting temperature and stability, we analyzed available data characterizing protein unfolding Gibbs free energies, ${\Delta G}^{0}$. Empirical ${\Delta G}^{0}$ values have now been obtained for a substantial number of proteins in *E. coli* and *H. sapiens* and are available in the ProTherm database (Kumar et al. 2006). This analysis revealed that Razban’s theoretical model is inconsistent with the strong empirical correlation for *E. coli* proteins between $T_{m}$ measurements from Leuenberger et al. (2017) and ${\Delta G}^{0}$ values in the ProTherm database (Pearson’s *r* = 0.69, *P*-value = 0.005; Spearman’s *r* = 0.62, *P*-value = 0.01).

**Fig. 1:**
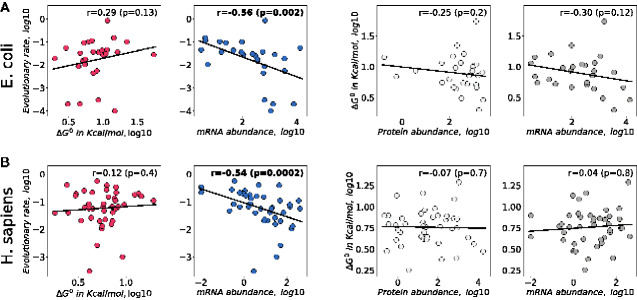
Correlations between experimentally measured ${\Delta G}^{0}$ values, protein abundances, mRNA abundances, and protein evolutionary rates. (*A*) *E. coli* (*n* = 28) and (*B*) *H. sapiens* (*n* = 42). The correlations between evolutionary rates and unfolding Gibbs free energies, ${\Delta G}^{0}$, are shown in the first figure column (red). The correlations between protein evolutionary rates and mRNA abundances are shown in the second column (blue). The correlations between ${\Delta G}^{0}$ and protein abundances are shown in the third column (light gray), and the correlations between ${\Delta G}^{0}$ and mRNA abundances are shown in the fourth column (dark gray). Solid lines represent the least square regression fits to the data. Spearman’s correlation coefficients and corresponding *P*-values are shown, significant correlations are highlighted in bold.

Importantly, the direct measure of protein stability, ${\Delta G}^{0}$, allows us to test the MAH regardless of an imperfect correlation between $T_{m}$ and ${\Delta G}^{0}$. For the subsets of proteins with available ${\Delta G}^{0}$ measurements, we were able to robustly reproduce a significant anticorrelation between evolutionary rates and mRNA abundances (fig. 1, blue; supplementary table 1, Supplementary Material online; Spearman’s r=-0.56, $P={2\times 10}^{-3}$, for *E. coli*; r=-0.54, $P={2\times 10}^{-4},$ for *H. sapiens*). Thus, the mechanisms that make highly expressed proteins evolve more slowly are likely to be reflected in the properties of these proteins. But contrary to the MAH predictions, we did not observe, for *E. coli* and *H. sapiens* proteins, a negative correlation between ${\Delta G}^{0}$ and evolutionary rates (fig. 1, red); we also did not observe positive correlations between ${\Delta G}^{0}$ and either protein or mRNA abundances (fig. 1, light and dark gray, respectively). When we restricted the ${\Delta G}^{0}$ analysis to include only monomeric proteins with two-state reversible (un)folding, the relationship between protein stability and abundance in *E. coli* became marginally significant but in the direction opposite to the one predicted by the MAH (Spearman’s r=-0.39, P=0.06; supplementary fig. 1, Supplementary Material online). This pattern may be due to well-known effects associated with the activity–stability tradeoff, that is, protein functional optimization which often leads to lower protein stability (Wang et al. 2002; Tokuriki et al. 2008; Knies et al. 2017). In summary, in agreement with the conclusions based on $T_{m}$ measurements from Leuenberger *et al.* (Plata and Vitkup 2018), the empirical ${\Delta G}^{0}$ data also do not provide any support for a major role of the MAH in explaining the variability of evolutionary rates across proteins.

In our view, the main caveat with Leuenberger *et al.* data set, in the context of testing the MAH, is not a poor correlation between $T_{m}$ and ${\Delta G}^{0}$. We previously demonstrated a substantial correlation (Pearson’s r=0.75, $P<10^{-20}$; Spearman’s r=0.64, $P<10^{-20}$) between these two characteristics of protein stability for measurements performed by the same research group (Plata and Vitkup 2018); and, as described above, we now also confirmed this correlation specifically for $T_{m}$ measurements from Leuenberger *et al.* Instead, intrinsic protein stability may simply not serve as a good proxy for protein aggregation propensity, which is likely to mediate misfolding toxicity. The protein melting temperatures obtained by Leuenberger et al. (2017) are based on data from limited proteolysis (LiP), which increases due to local protein unfolding triggered by higher temperatures; below, we refer to these melting temperature measurements using the term $T_{m}^{\mathrm{LiP}}$. An alternative method, developed by Savitski et al. (2014), uses protein aggregation (Agg) as a proxy of unfolding. This method estimates melting temperatures by quantifying proteins’ concentrations in soluble cellular fractions as a function of temperature; we refer to these melting temperature measurements using the term $T_{m}^{\mathrm{Agg}}$. Because $T_{m}^{\mathrm{Agg}}$ is likely to be a good measure of protein propensity to aggregate and therefore to cause misfolding toxicity, we analyzed next its correlations with protein abundance and evolutionary rates.

**Fig. 2:**
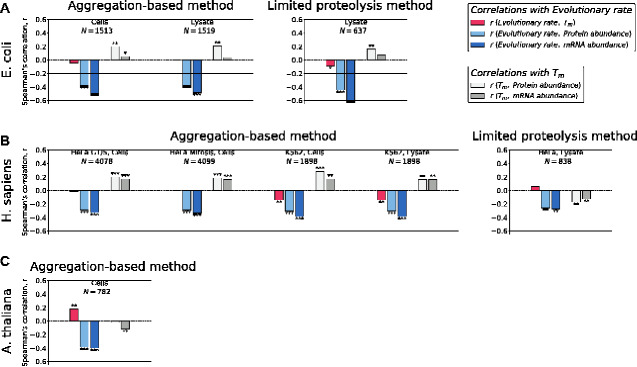
Correlations between genome-wide melting temperatures, protein abundances, mRNA abundances, and protein evolutionary rates. (A) *E. coli*, (*B*) *H. sapiens*, and (*C*) *A. thaliana.* Bar plots show the values of the Spearman’s correlation coefficients between evolutionary rates and melting temperatures (red), between evolutionary rates and protein abundances (light blue), between evolutionary rates and mRNA abundances (dark blue), between melting temperatures and protein abundances (light gray), and between melting temperatures and mRNA abundances (dark gray). Different experimental methodologies used to measure $T_{m}$ and different sample types are indicated above corresponding figure panels for *E. coli* and *H. sapiens*. Numbers of proteins in the analyzed data sets are also shown; in each data set we kept only proteins for which all four parameters (melting temperature, protein abundance, mRNA abundance, and evolutionary rate) are known. Asterisks above and below bars indicate significance levels: *P-value < 0.05, **P-value < 0.001, ***P-value < 10^−25^.

To analyze the potential effects of protein aggregation on protein evolution, we used the $T_{m}^{\mathrm{Agg}}$ data for approximately 1,500 *E. coli* proteins based on measurements performed in cells and natural cellular lysates (Mateus et al. 2018). Interestingly, we found that in both data sets $T_{m}^{\mathrm{Agg}}$ significantly correlated with protein abundances (fig. 2*A*, light gray; supplementary table 2, Supplementary Material online; Spearman’s r=0.20, $P<10^{-14}$, for cells; r=0.21, $P<10^{-15}$, for cellular lysates). However, we observed no significant correlations between $T_{m}^{\mathrm{Agg}}$ and evolutionary rates (fig. 2*A*, red). $T_{m}^{\mathrm{Agg}}$ were also independently measured in two different *H. sapiens* cell lines: HeLa cells (Becher et al. 2018), where the $T_{m}^{\mathrm{Agg}}$ for approximately 4,000 proteins were obtained for intact cells in different cell-cycle stages (G1/S transition and mitosis), and K562 chronic myeloid leukemia cells (Savitski et al. 2014), where the $T_{m}^{\mathrm{Agg}}$ for approximately 2,000 proteins were obtained for intact cells and cellular lysates. Analyzing these measurements, we found that across all human data sets $T_{m}^{\mathrm{Agg}}$ also positively correlated with protein abundances (fig. 2*B*, light gray; Spearman’s $r=0.20,\;P<10^{-38}$; r=0.19, $P<10^{-32}$; r=0.29, $P<10^{-36}$; and r=0.16, $P<10^{-11}$). For the K562 data sets, $T_{m}^{\mathrm{Agg}}$ values were also negatively correlated with protein evolutionary rates (fig. 2*B*, red; Spearman’s r=-0.14, $P<10^{-9}$, for both cells and lysate). Finally, $T_{m}^{\mathrm{Agg}}$ data were also obtained for approximately 800 proteins in *Arabidopsis thaliana* (Volkening et al. 2019). For that data set, we did not find any correlation of $T_{m}^{\mathrm{Agg}}$ with protein abundances (fig. 2*C*, light gray) and the correlation with evolutionary rates was significant, but positive (fig. 2*C*, red; Spearman’s r=0.18, $P<10^{-6}$).

What fraction of the molecular clock variance is explained by the observed correlations with $T_{m}^{\mathrm{Agg}}$? According to the MAH, avoiding cytotoxicity is a major driver of the variability in protein evolutionary rates. However, our analyses demonstrate that the anticorrelation between $T_{m}^{\mathrm{Agg}}$ and evolutionary rates (fig. 2, red) is significant only in two (out of seven) data sets, and even in these two, $T_{m}^{\mathrm{Agg}}$ explain only ~2% of the variance in evolutionary rates across proteins. For comparison, mRNA abundance explains about an order of magnitude higher fraction of the evolutionary rate variance (fig. 2, dark blue), that is, approximately 15% for the same subset of proteins. Furthermore, due to weak correlations between $T_{m}^{\mathrm{Agg}}$ and evolutionary rates, we found that the anticorrelations between mRNA abundances and evolutionary rates do not substantially decrease after controlling for $T_{m}^{\mathrm{Agg}}$ (e.g., Spearman’s $r_{\mathrm{Ev}.\mathrm{Rate}-\mathrm{mRNA}}=-0.38$ and corresponding Spearman’s partial $r_{\mathrm{Ev}.\;\mathrm{Rate}-\mathrm{mRNA}|T_{m}^{\mathrm{Agg}}\;}=-0.37$ for *H. sapiens* K562 cells, for the data set with the strongest effects of $T_{m}^{\mathrm{Agg}}$).

**Fig. 3:**
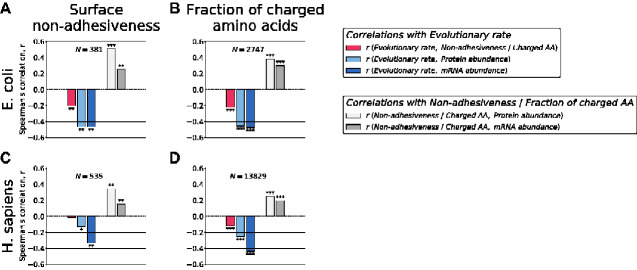
Correlations between protein surface nonadhesiveness, the fraction of charged amino acids, protein abundances, mRNA abundances, and protein evolutionary rates. (*A*, *B*) *E. coli* and (*C*, *D*) *H. sapiens*. Bar plots in (*A*) and (*C*) show the values of Spearman’s correlation coefficients between evolutionary rates and protein surface nonadhesiveness (red), between protein surface nonadhesiveness and protein abundance (light gray), and between protein surface nonadhesiveness and mRNA abundance (dark gray). Bar plots in (*B*) and (*D*) show the values of Spearman’s correlation coefficients between evolutionary rates and the fraction of charged amino acids (red), between the fraction of charged amino acids and protein abundance (light gray), and between the fraction of charged amino acids and mRNA abundance (dark gray). In all panels the values of Spearman’s correlation coefficients between evolutionary rates and protein abundances (light blue), and between evolutionary rates and mRNA abundances (dark blue) are also shown. Numbers of proteins in the analyzed data sets are indicated, in each data set we kept only proteins for which all four parameters (protein abundance, mRNA abundance, evolutionary rate and surface nonadhesiveness or the fraction of charged amino acids) are known. Asterisks above and below bars represent significance levels: *P-value < 0.05, **P-value < 0.001, ***P-value < 10^−25^.

To put the observed correlations with $T_{m}^{\mathrm{Agg}}$ into perspective, we note that multiple other protein properties, such as the fraction of charged amino acids (Plata et al. 2010), protein solubility (Plata et al. 2010), surface stickiness (Levy et al. 2012), and the number of protein–protein interaction partners (Yang et al. 2012), have been shown to correlate with protein abundance. In *E. coli* (Plata et al. 2010; Levy et al. 2012), *Saccharomyces cerevisiae* (Levy et al. 2012; Yang et al. 2012), and *H. sapiens* (Levy et al. 2012), changes of these properties for abundant proteins likely help to alleviate deleterious effects of nonfunctional interactions and binding. For example, protein surface nonadhesiveness or the fraction of charged amino acids correlate positively, and with similar strength as $T_{m}^{\mathrm{Agg}}$, with protein abundances (fig. 3, light gray), and negatively with evolutionary rates (fig. 3, red, supplementary tables 3 and 4, Supplementary Material online). However, the ability of all these protein characteristics to explain the variability of evolutionary rates is modest compared to that of mRNA abundance (fig. 3, dark blue). Notably, protein surface nonadhesiveness, the fraction of charged amino acids, and effects quantified by $T_{m}^{\mathrm{Agg}}$ likely represent complementary sources of constraints, as these properties do not correlate strongly with each other (supplementary table 5, Supplementary Material online).

## Discussion

In this work we continued to test the MAH using various empirical data sets, and the presented results agree with and extend our previous conclusions (Plata et al. 2010; Plata and Vitkup 2018). The original MAH hypothesis was motivated, at least in part, by several studies demonstrating that protein evolutionary rates correlate only weakly with the fitness effects arising due to complete gene deletions (Hurst and Smith 1999; Hirsh and Fraser 2001; Pal et al. 2003). However, it is important not to conflate the effects associated with complete gene deletions and the level of overall protein sequence constraints which directly affect the rate of molecular clock. As discussed previously (Cherry 2010; Zhang and Yang 2015), protein sequence constraints primarily reflect selection against small fitness effects of single mutations rather than fitness loss associated with null mutations. Analogous observations have been made in different contexts. For example, although close yeast gene duplicates provide good buffering for complete knockouts of one homolog, individual amino acid mutations in close duplicates are actually more deleterious compared with mutations in genes with distant homologs (Plata and Vitkup 2014). 

A key question that the MAH was supposed to resolve is the nature of increased protein sequence constraints of highly abundant proteins. The original MAH (Drummond et al. 2005; Drummond and Wilke 2008) and its multiple extensions (Yang et al. 2010; Serohijos et al. 2012) proposed that these constraints primarily originate from the increased stability of highly expressed proteins. But, based either on direct measurements of protein stability (${\Delta G}^{0}$) or on its various proxies ($T_{m}^{\mathrm{LiP}}$, $T_{m}^{\mathrm{Agg}}$, see supplementary table 6, Supplementary Material online), we do not find support for this key MAH prediction. Proteins clearly need to be stable to perform their molecular and biological function, and maintaining protein stability does constrain sequence evolution (Dill and Bromberg 2012). Multiple deep mutational scanning experiments demonstrate that fitness effects of substitutions correlate with their $\Delta {\Delta G}^{0}$, that is, destabilizing mutations tend to be more deleterious (Jacquier et al. 2013; Firnberg et al. 2014; Sarkisyan et al. 2016). Nevertheless, overall stability constraints are similar for different proteins, and differences in protein stability do not seem to play a major role in explaining the *variability* of evolutionary rates across cellular proteins. There is no paradox here, and this conclusion is in fact consistent with multiple empirical and biophysical data beyond the $T_{m}$ and ${\Delta G}^{0}$ measurements. For example, it was demonstrated that the strength of the correlation between evolutionary rates and mRNA abundances is similar for sites with different contributions to protein stability, such as surface sites and sites in protein cores (Yang et al. 2012). Moreover, increasing protein stability beyond a certain threshold is not evolutionary advantageous, and may be generally detrimental to fitness, as demonstrated by multiple known examples of stability–activity trade-offs in proteins (Wang et al. 2002; Tokuriki et al. 2008; Knies et al. 2017). If effects associated with misfolding become harmful, for example, due to significantly increased burden of transcriptional (Goldsmith and Tawfik 2009) or translational (Bratulic et al. 2015) errors, proteins are quickly stabilized by fixation of several mutations (Goldsmith and Tawfik 2009; Bratulic et al. 2015) without substantial further constraints on the corresponding protein sequence.

Across the *E. coli* and *H. sapiens* data sets we analyzed, $T_{m}^{\mathrm{Agg}}$ correlates better with protein abundances, whereas evolutionary rates correlate more strongly with mRNA abundances (fig. 2). Moreover, there is very little mRNA-independent, that is, protein-specific, contribution to the correlations with evolutionary rates. This provides another strong argument against the MAH which predicts that protein misfolding and therefore protein abundance should be the main driver of the varaibility of evolutionary constraints. For the entire *H. sapiens* proteome, Spearman’s correlation between mRNA abundance (Mele et al. 2015) and evolutionary rate is 0.44, while the correlation between protein abundance (Wang et al. 2015) and evolutionary rate is 0.26. Based on partial correlations, protein abundance explains ~1% extra variance of evolutionary rates in addition to mRNA abundance; similarly, in *E. coli* the independent contribution of protein abundance to the variance of evolutionary rates is ~5%. These results provide further evidence that different biological mechanisms may be driving constraints related to protein aggregation and misinteractions and those responsible for the substantial variability of protein evolutionary rates.

Direct experimental measurements demonstrated that deleterious mutations reduce fitness primarily due to changes in protein function, rather than due to destabilization-induced changes in protein abundance (Firnberg et al. 2014). Analyzing long-term protein evolution, we also recently showed that *functional optimality*, that is, the conservation of protein sequence and 3D structure necessary for efficient protein function, is a substantially stronger evolutionary constraint than the requirement to simply maintain folded protein stability (Konate et al. 2019). Recently, the fraction of mutations leading to deleterious effects through all possible nonfunctional mechanisms, referred to as collateral effects, was estimated to be approximately 40% for the TEM-1 protein in *E. coli* (Mehlhoff et al. 2020). This result also suggests that collateral effects are unlikely to dominate protein evolutionary constraints, at least for the vast majority of bacterial proteins. Based on the ratio of amino acid changing to synonymous substitutions in bacteria, $K_{a}/K_{s}$ ∼ 0.05–0.1 (Koonin 2012), the fraction of bacterial amino acid changing mutations that are rejected in evolution is approximately 0.9–0.95. Therefore, functional effects play a larger role in purifying selection even under the assumption that collateral (non-functional) mechanisms dominate functional mechanisms for all mutations with non-zero collateral effects. And this assumption is quite unlikely as protein sites of collateral mutations substantially overlap with functionally sensitive sites, and collateral effects are often smaller in magnitude compared with functional effects (Stiffler et al. 2015; Mehlhoff et al. 2020). All these results suggest that the diversity of protein evolutionary rates may be more related to functional effects of substitutions rather than effects associated with protein stability. Evolutionary models and corresponding computational simulations (Cherry 2010; Gout et al. 2010) also demonstrated the plausibility that functional optimization, which allows cells to minimize the cost of gratuitous protein expression, may be responsible for higher level of sequence constraints of abundant proteins.

Different models of protein sequence evolution emphasized different costs of protein production. Therefore, the primary origin of protein expression costs is another important evolutionary question related to the MAH. In the original MAH hypothesis this cost was proposed to arise from protein misfolding induced by translational errors (Drummond et al. 2005; Drummond and Wilke 2008), and this cost was later extended to error-free misfolding (Yang et al. 2010). We note that several previous experimental studies did not find substantial costs associated with protein misfolding (Plata et al. 2010; Kafri et al. 2016). By overexpressing pairs of close yeast duplicates, evolving at different rates, a recent study by Biesiadecka et al. (2020) also did not find any evidence of substantial contribution of costs associated with translation-induced misfolding. Although the study by Geiler-Samerotte et al. (2011) is often viewed as supporting a major role of the MAH (Zhang and Yang 2015), the critical analysis of the results reported in that study also suggest a smaller cost of translation-induced misfolding compared with other costs associated with protein production. Specifically, Geiler-Samerotte et al. showed that the gratuitous overexpression of a protein with multiple destabilizing substitutions leads to deleterious fitness effects which are approximately three times higher than the expression cost of the wild-type protein. However, mistranslation errors are present in only approximately 15% of proteins (Drummond and Wilke 2008; Yang et al. 2010), and even a smaller fraction of proteins contains multiple translation-induced mutations or mutations with substantial destabilizing effects (e.g., only ∼20% of mutations have $\Delta {\Delta G}^{0}<-2\;$kcal/mol) (Nisthal et al. 2019). Therefore, the overall cost (per protein) of translation-induced misfolding is substantially smaller than the cost of protein production. The same conclusion can be extended to error-free misfolding based on the estimate that it contributes only 5–20% extra misfolding events compared with misfolding arising from translational errors (Yang et al. 2010).

Finally, although the feasibility of a dominant MAH contribution was suggested by computational simulations (Drummond and Wilke 2008; Yang et al. 2010; Serohijos et al. 2012), biology is an empirical science, and the fidelity of proposed hypotheses should be ultimately determined by their agreement, or lack thereof, with available experimental data. An obvious weakness of aforementioned simulations is that they only considered effects associated with protein stability and interactions. Thus, they demonstrated the MAH feasibility but could not evaluate the relative importance of other biological and functional effects. While it is often possible to invoke sophisticated noise and error models to explain the absence of expected observations (Razban 2019), based on the preponderance of available evidence the MAH is unlikely to play any major role in mediating a strong negative correlation between protein abundances and evolutionary rates. Major roles are also unlikely for several other mechanisms, such as effects associated with increased protein solubility or avoidance of nonfunctional interactions (Plata et al. 2010; Yang et al. 2012). Most importantly, the search for the main factors contributing to the substantial variability of evolutionary rates across proteins must continue.

## Materials and Methods

The protein stability data were obtained from the ProTherm database (Kumar et al. 2006), which have been recently moved to the ProtaBank (Wang et al. 2019) and are available at https://github.com/protabit/protherm-conversion. We considered unfolding Gibbs free energies, ${\Delta G}^{0}$, for wild-type proteins measured at pH values between 4 and 9, and for temperatures between 10 and 50 °C. Proteins in all oligomeric states and with any (un)folding dynamics were used in figure 1. Only monomers that exhibit two-state reversible (un)folding were used in supplementary figure 1, Supplementary Material online. For each protein with more than one available measurement, the values were averaged over different experimental conditions and measurements performed by different research groups. Raw ${\Delta G}^{0}$ values for *E. coli* and *H. sapiens* extracted from ProTherm are available in supplementary table 7, Supplementary Material online.

We used the rate of nonsynonymous substitutions, $K_{a}$, as a measure of protein evolutionary rate. $K_{a}$ values for *E. coli*, *H. sapiens*, and *A. thaliana* were calculated using the PAML package (Yang 1997) relative to *Salmonella enterica*, *Mus musculus*, and *Brassica oleracea*. Orthologs were identified as bidirectional best hits in pairwise local alignments calculated with Usearch (Edgar 2010). We considered for analysis only protein pairs for which corresponding alignments covered at least 70% of the shortest protein length.

Protein abundance data for all species were obtained from the whole-organism integrated data sets available in the PaxDB v.4 database (Wang et al. 2015). mRNA abundances data for *E. coli* were obtained from McClure et al. (2013). mRNA abundances from the brain frontal cortex (Mele et al. 2015) was used for *H. sapiens*, as it was demonstrated that mRNA expression in this tissue has the highest correlation with protein evolutionary rates (Drummond and Wilke 2008). mRNA abundances from the germinating seed was used for *A. thaliana* (Klepikova et al. 2016).

Protein surface nonadhesiveness was obtained from Levy et al. (2012). Specifically, this measure equals the negative sum of amino acid stickiness scores (Levy et al. 2012) across sequence sites located on protein surfaces based on corresponding protein 3D structures (Levy 2010).

## Supplementary Material

Supplementary data are available at *Genome Biology and Evolution* online.

## Supplementary Material

evab006_Supplementary_Data
