# Supplementary material for: The Relationship between the Misfolding Avoidance Hypothesis and Protein Evolutionary Rates in the Light of Empirical Evidence
Source: Genome Biol Evol. 2021 Jan 11;13(2):evab006. doi: 10.1093/gbe/evab006 (PMC7874998; doi:10.1093/gbe/evab006)
Supplement: evab006_Supplementary_Data [file evab006_supplementary_data.zip › 641_Supplementary Figure and Table legends.docx]

**Supplementary Figure 1. Correlations between** **experimentally measured** $\boldsymbol{\Delta G}^{\boldsymbol{0}}$ **values, protein abundances, mRNA abundances, and protein evolutionary rates for monomers with 2-state reversible (un)folding**

**A.** *E. coli* (n=24) and **B.** *H. sapiens* (n=38)*.* The correlations between evolutionary rates and unfolding Gibbs free energies, ${\Delta G}^{0}$, are shown in the first figure column (red). The correlations between protein evolutionary rates and mRNA abundances are shown in the second column (blue). The correlations between $\Delta G$ and protein abundances are shown in the third column (light grey), and the correlations between ${\Delta G}^{0}$ and mRNA abundances are shown in the fourth column (dark grey). Solid lines represent the least square regressions fitted to the data. Spearman’s correlation coefficients and corresponding p-values are shown, significant correlations are highlighted in bold. Only ${\Delta G}^{0}$ values for monomers which exhibit 2-state reversible (un)folding are included in this analysis.

**Supplementary Table S1**

Correlations between experimentally measured ${\Delta G}^{0}$ values, protein abundances, mRNA abundances, and protein evolutionary rates

**Supplementary Table S2**

Correlations between genome-wide melting temperatures, protein abundances, mRNA abundances, and protein evolutionary rates

**Supplementary Table S3**

Correlations between protein surface non-adhesiveness, protein abundances, mRNA abundances, and protein evolutionary rates

**Supplementary Table S4**

Correlations between fractions of charged amino acids, protein abundances, mRNA abundances, and protein evolutionary rates

**Supplementary Table S5**

Correlations between protein surface non-adhesiveness, fractions of charged amino acids, and melting temperatures

**Supplementary Table S6**

Correlations between different proxies of protein stability (${\Delta G}^{0}$, $T_{m}^{LiP}$,$T_{m}^{Agg}$)

**Supplementary Table S7**

${\Delta G}^{0}$ values for *E. coli* and *H. sapiens* extracted from ProTherm

**Supplementary Table S8**

Data on evolutionary rates, mRNA abundances, protein abundancies, ${\Delta G}^{0}$, melting temperatures, surface non-adhesiveness, and fractions of charged amino acids for *E. coli*.

**Supplementary Table S9**

Data on evolutionary rates, mRNA abundances, protein abundancies, ${\Delta G}^{0}$, melting temperatures, surface non-adhesiveness, and fractions of charged amino acids for *H. sapiens*.

**Supplementary Table S10**

Data on evolutionary rates, mRNA abundances, protein abundancies, melting temperatures, and fractions of charged amino acids for *A. thaliana*.
