# Supplementary figures and images for: The Relationship between the Misfolding Avoidance Hypothesis and Protein Evolutionary Rates in the Light of Empirical Evidence
Source: Genome Biol Evol. 2021 Jan 11;13(2):evab006. doi: 10.1093/gbe/evab006 (PMC7874998; doi:10.1093/gbe/evab006)

**A****E. coli**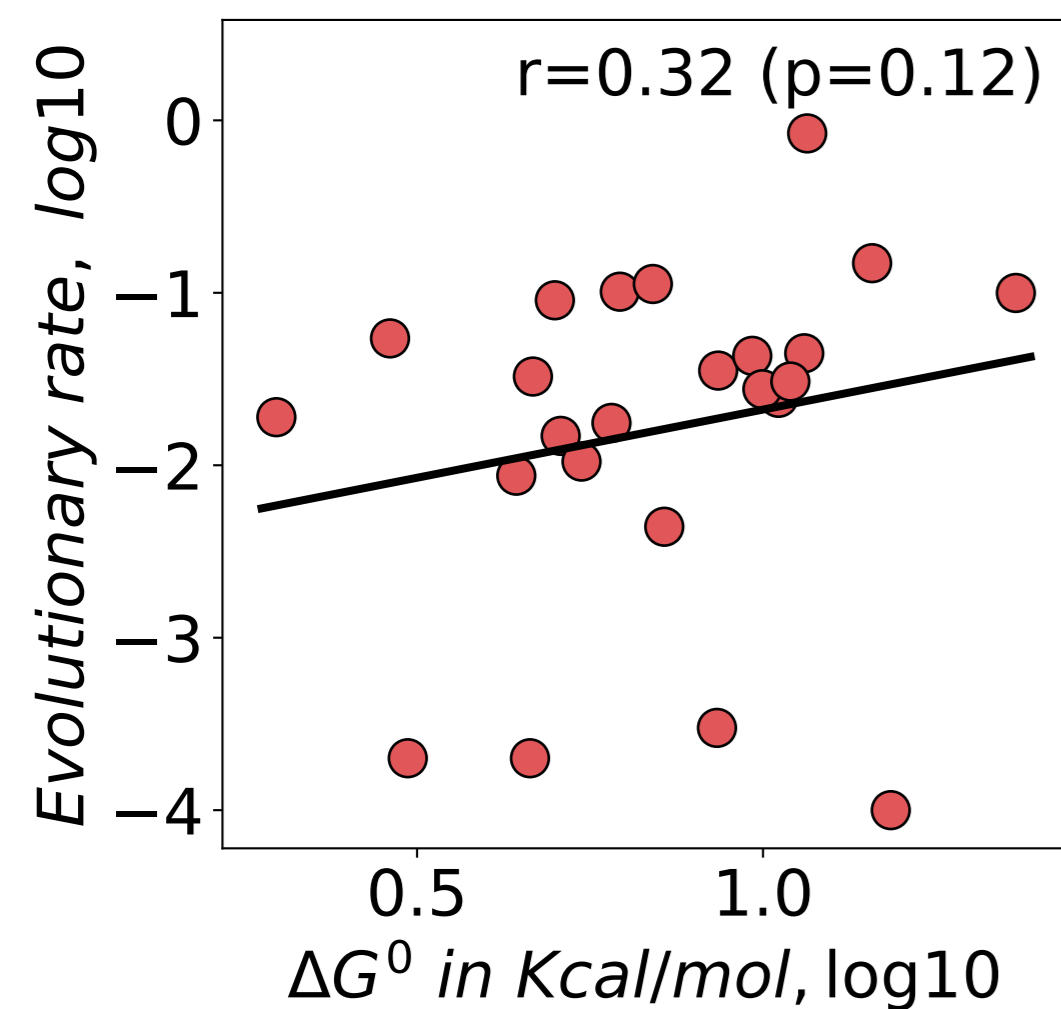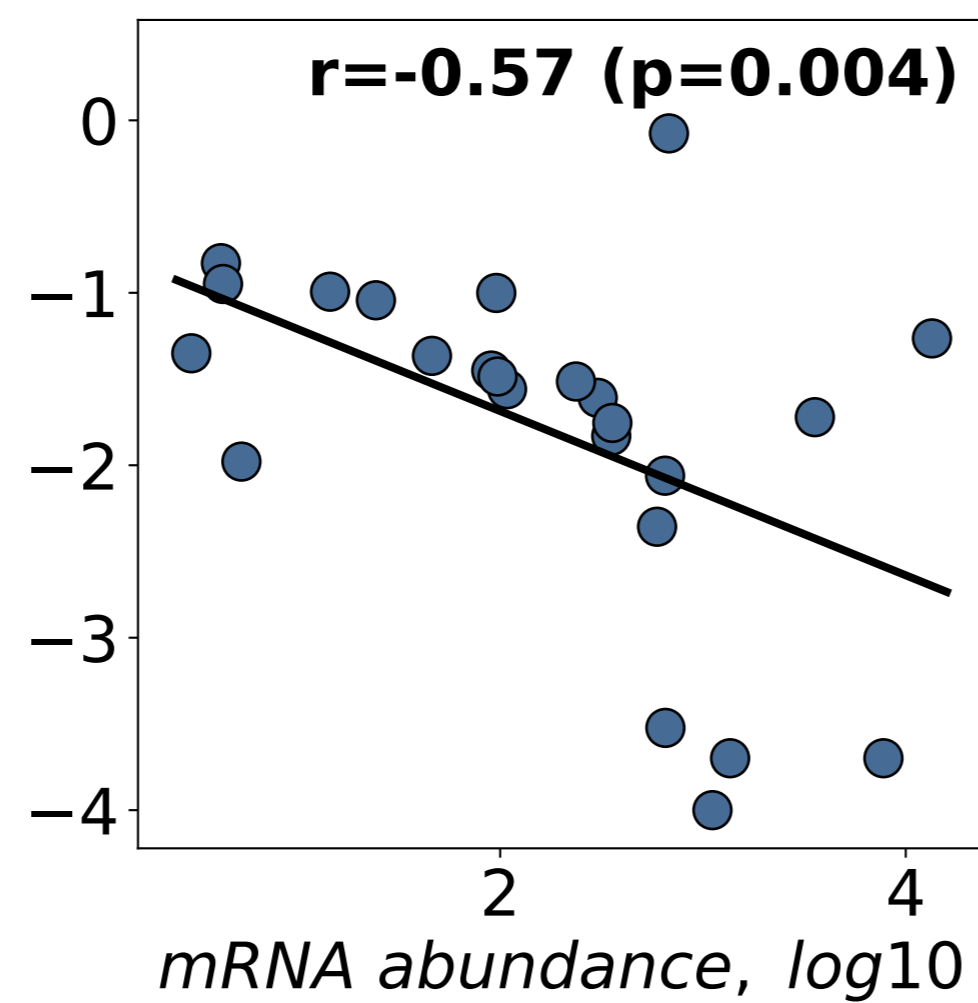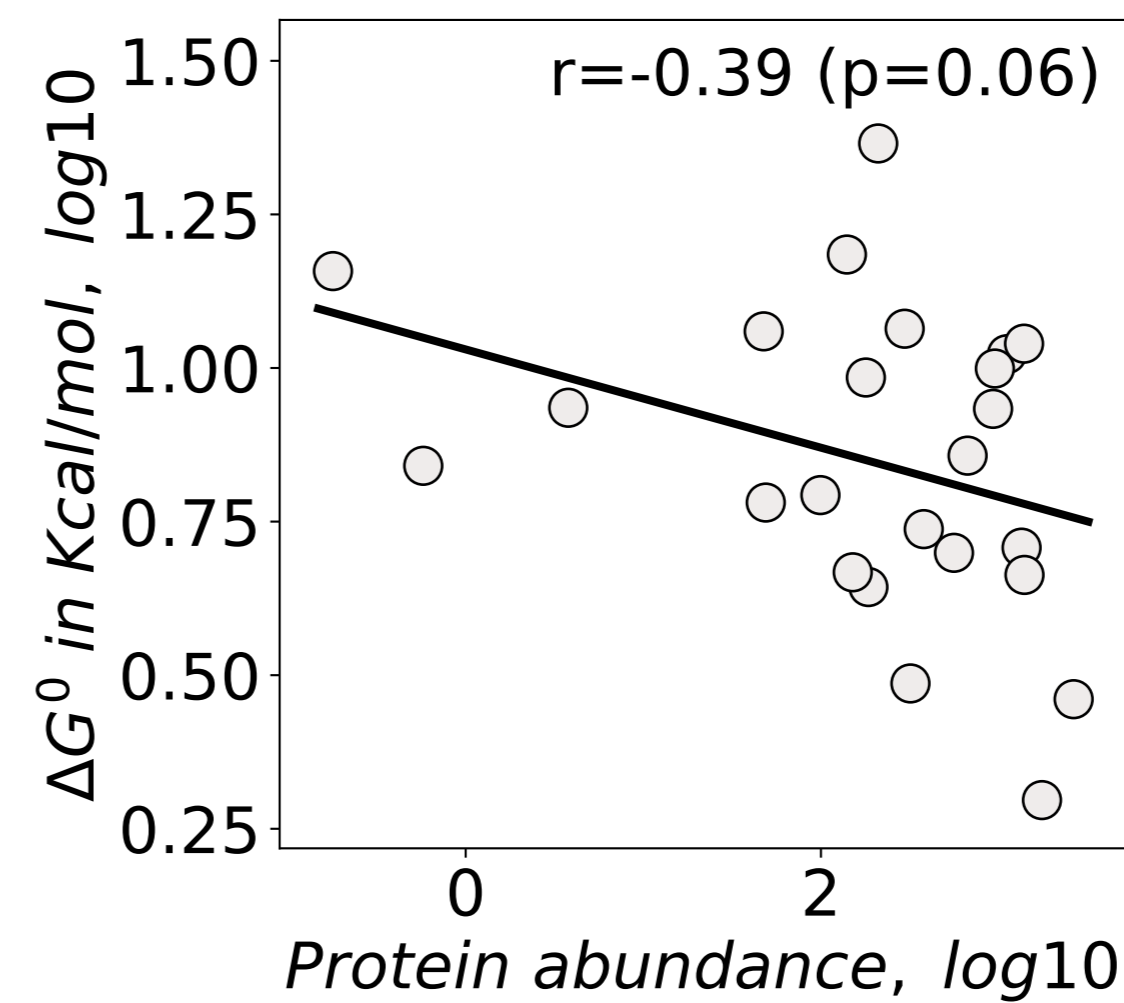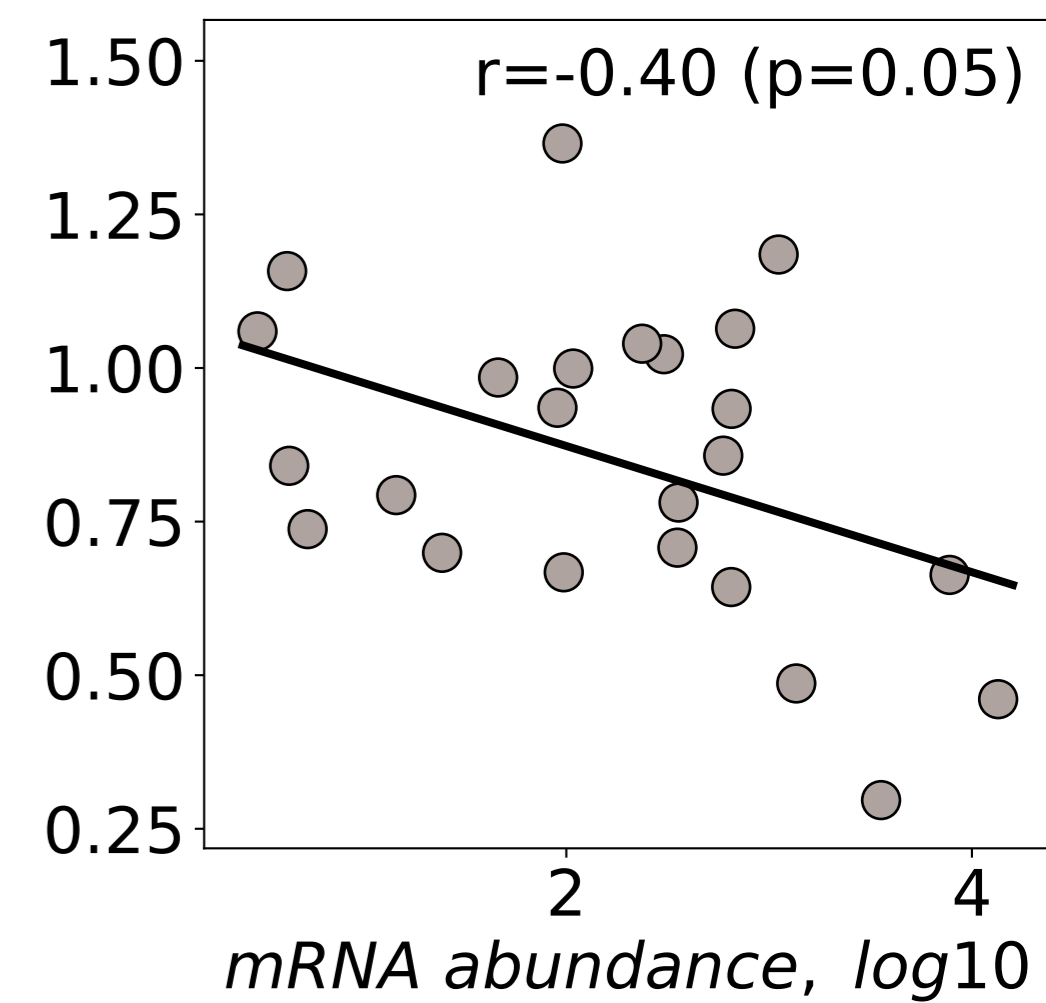**B****H. sapiens**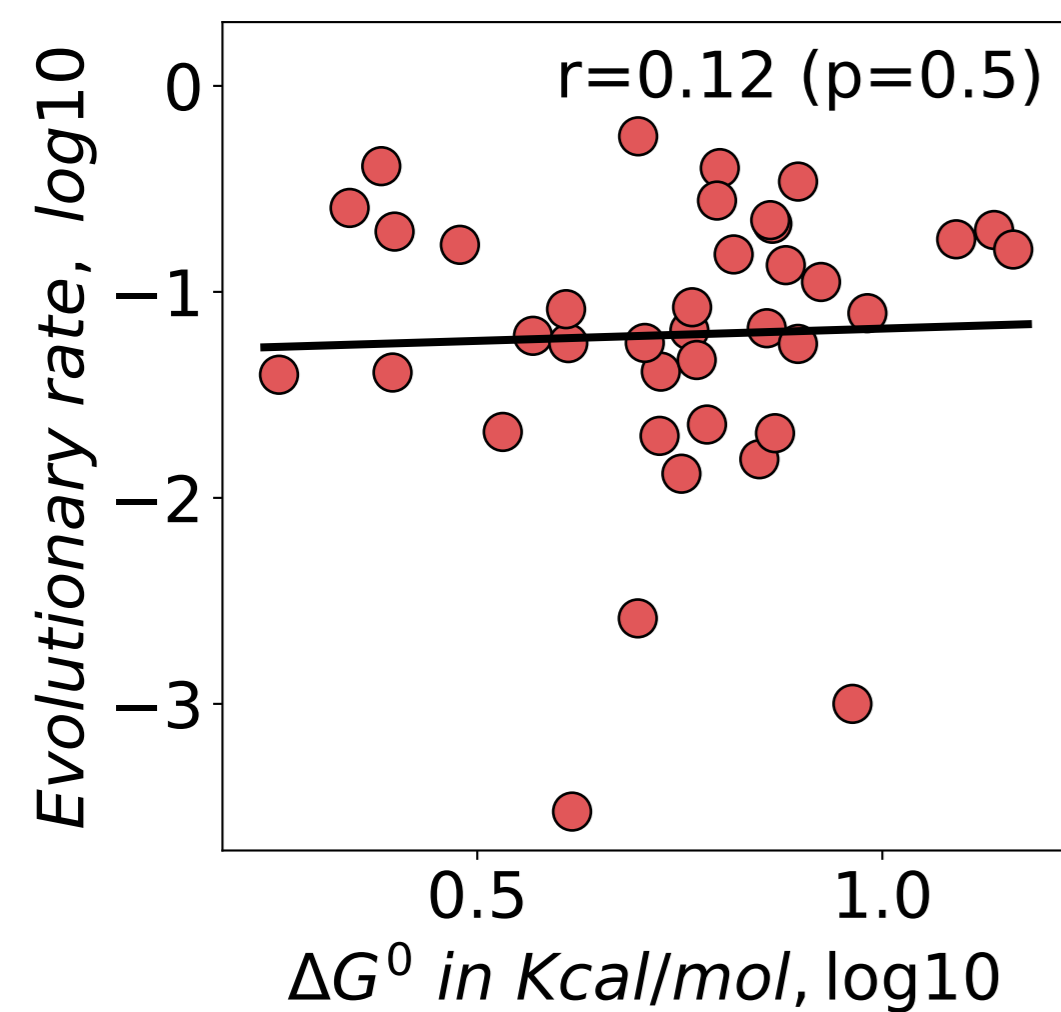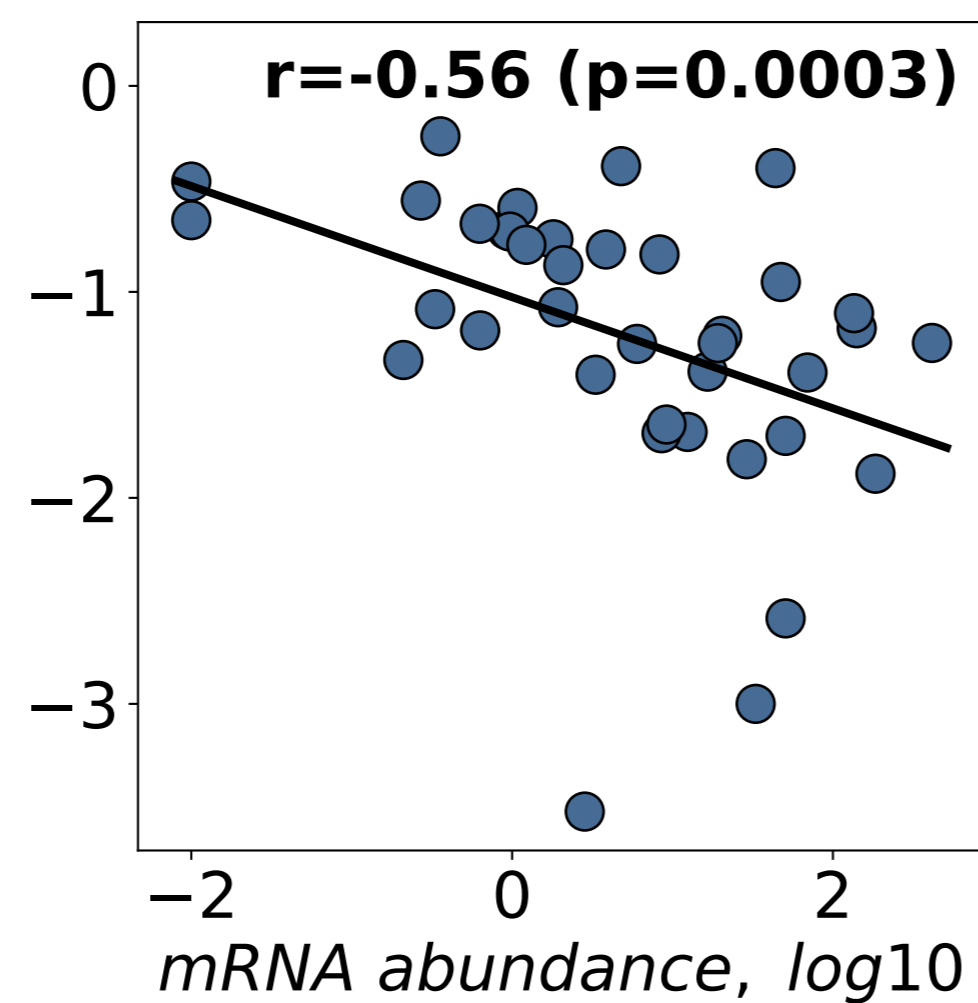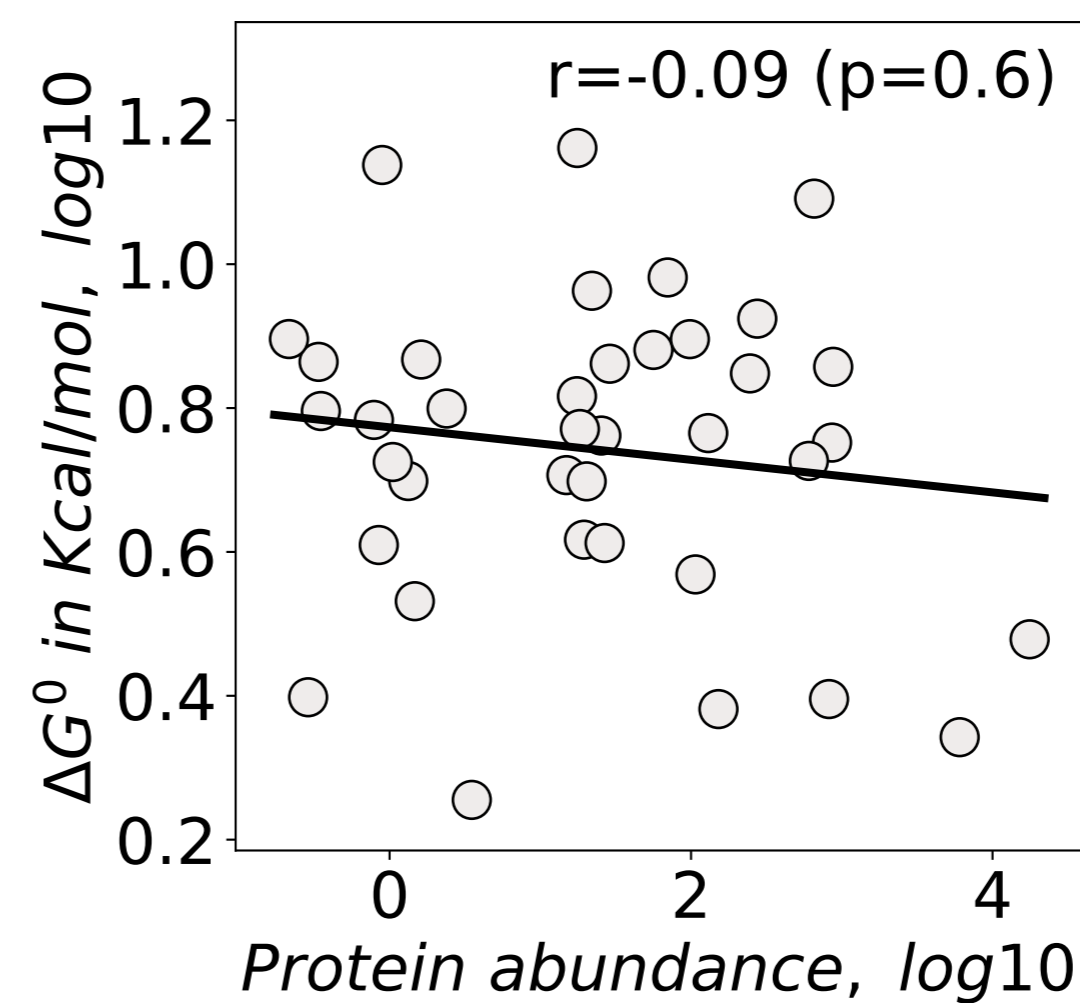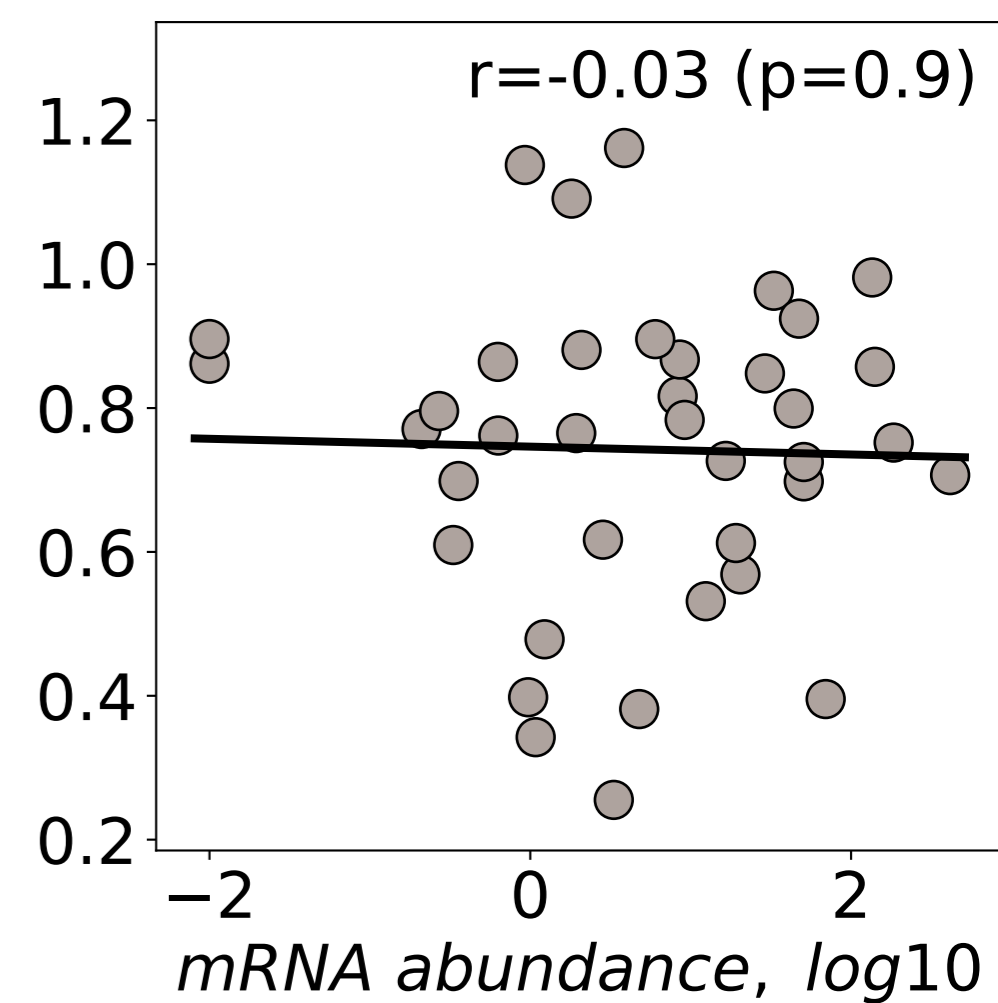

Supplement: evab006_Supplementary_Data [file evab006_supplementary_data.zip › SupplimentaryFigure1.pdf]
